# 1384. Practice Variations in Pre-Hematopoietic Stem Cell Transplantation Infectious Disease Evaluation

**DOI:** 10.1093/ofid/ofab466.1576

**Published:** 2021-12-04

**Authors:** Liam S Conway-Pearson, Jennifer Pisano, Roberto A Sica, Margaret E McCort

**Affiliations:** 1 Montefiore Medical Center, New York, New York; 2 University of Chicago, Chicago, IL; 3 Montefiore Medical Center / Albert Einstein College of Medicine, Bronx, New York

## Abstract

**Background:**

Hematopoietic stem cell transplantation (HCT), and other forms of cellular therapies such as chimeric antigen receptor T cell therapy (CAR-T), while of critical therapeutic value, confers significant, long-term risk of infectious complications. Recipients would benefit from evaluation by infectious disease (ID) specialists. However, amidst many existing guidelines from ID and oncology societies, pre-transplant ID evaluation and management practices vary across US institutions. To better understand these variations and identify targets for standardization, we conducted a survey of ID and oncology providers at transplant centers in the US.

**Methods:**

A 38-question, anonymous, voluntary, online survey was distributed via Google Forms to a professional organization e-mail list of ID providers as well as to followers of relevant Twitter accounts. Responses were collected and analyzed.

**Results:**

A total of 51 responses were received, the majority of which (68.6%) came from ID providers. 60.8% of respondents worked at healthcare facilities with over 500 beds. 43 respondents (84.3%) reported that their center performed autologous and allogeneic HCT as well as CAR-T. 56.8% of CAR-T centers use a standardized template, compared to 70.8% of those providing HCT. For allogeneic HCT centers, 8% reported that no ID evaluation is offered; 34% reported that it is offered “sometimes.” Practices varied for treatment of latent tuberculosis infection prior to HCT: 26.5% treat “All the time;” 10.2% treat “Very rarely.” In assessing risk factors, only 63% and 54% identified HIV infection and healthcare occupation, respectively, as epidemiologic risk factors for tuberculosis infection. 59.2% answered that < 10% of patients are screened for Strongyloides. Only 5 respondents reported universal *Strongyloides* screening prior to transplant. COVID-19 vaccination for family is recommended “Always” by 95.5% of respondents. 25% have offered influenza vaccination to family through the transplant clinic.

Table 1

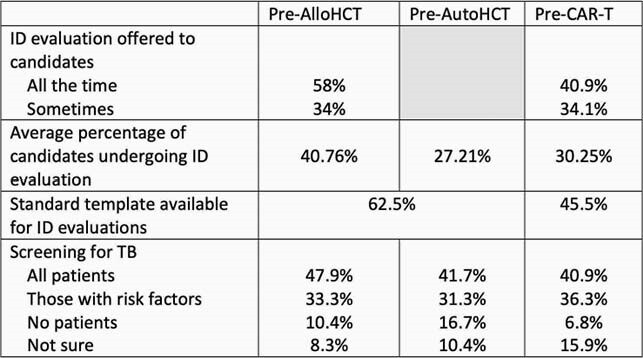

Differences in ID evaluation practices by type of cellular therapy candidate.

Table 2

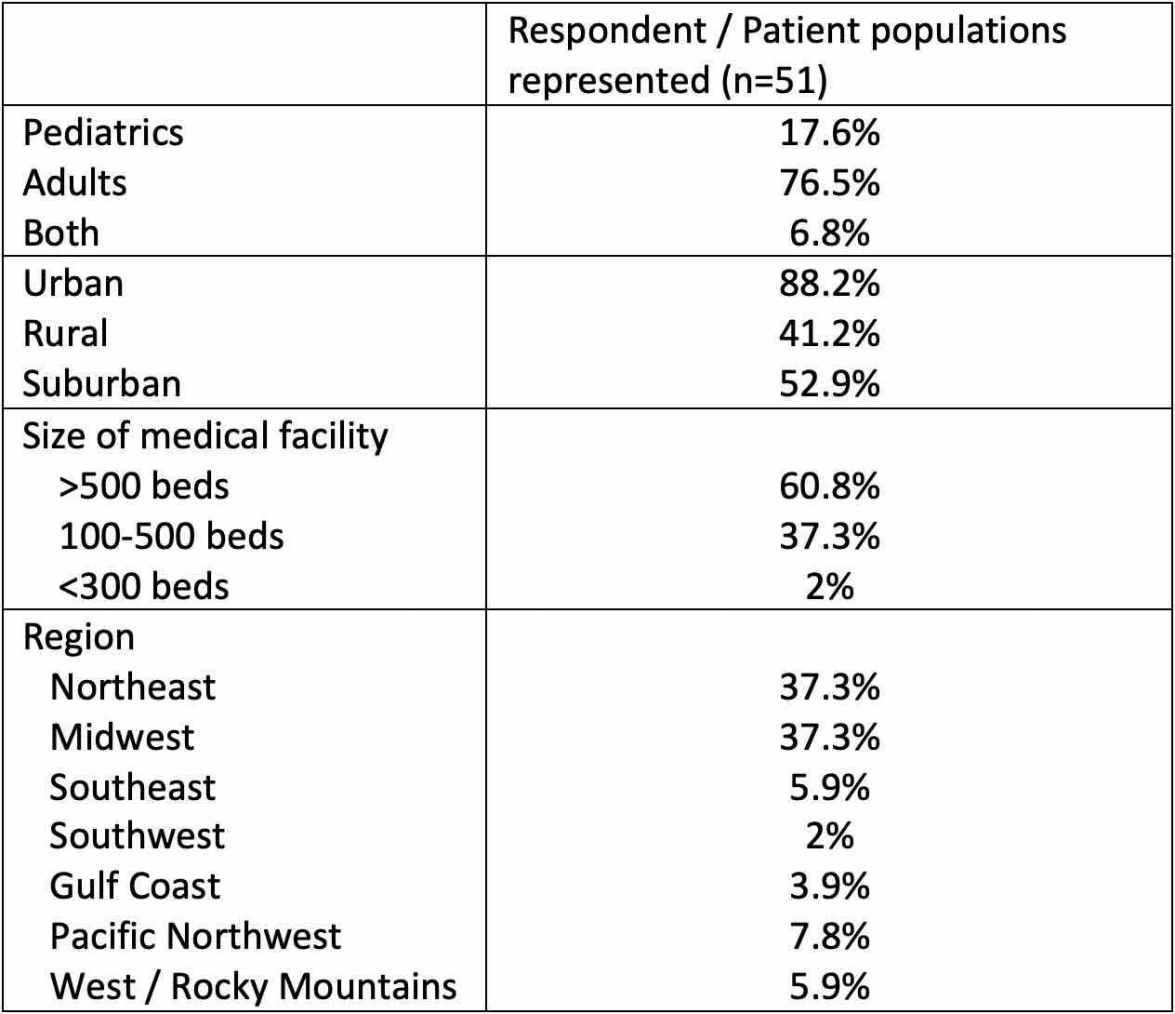

Characteristics of survey respondents.

**Conclusion:**

Practices around pre-HCT infectious disease evaluation and management are heterogenous among the centers surveyed. The adoption of standardized screening for and management of infectious diseases in this patient population would likely be beneficial.

**Disclosures:**

**All Authors**: No reported disclosures

